# Altered Gene Expression in the Testis of Infertile Patients with Nonobstructive Azoospermia

**DOI:** 10.1155/2021/5533483

**Published:** 2021-06-09

**Authors:** Zhiqiang Wang, Zhongjun Ding, Yan Guan, Chunhui Liu, Linjun Wang, Wensheng Shan, Jie Yang

**Affiliations:** ^1^Gansu Provincial Maternal and Children Health Care Hospital, China; ^2^Affiliated Hospital of Gansu University of Chinese Medicine, China

## Abstract

**Background:**

The molecular mechanism of nonobstructive azoospermia (NOA) remains unclear. The aim of this study was to identify gene expression changes in NOA patients and to explore potential biomarkers and therapeutic targets.

**Methods:**

The gene expression profiles of GSE45885 and GSE145467 were collected from the Gene Expression Omnibus (GEO) database, and the differences between NOA and normal spermatogenesis were analyzed. Enrichment analysis was performed to explore biological functions for common differentially expressed genes (DEGs) in GSE45885 and GSE145467. Coexpression analysis of DEGs in GSE45885 was performed, and two modules with the highest correlation with NOA were screened. Key genes were then screened from the intersection genes of the two modules and common DEGs and PPI network. The expression of key genes was validated by quantitative real-time polymerase chain reaction (qRT-PCR) experiments. Finally, through miRTarBase, miRDB, and RAID, the miRNAs were predicted to regulate key genes, respectively.

**Results:**

A total of 345 common DEGs were identified and they were mainly related to spermatogenesis, insulin signaling pathway. Coexpression analysis of DEGs in GSE45885 yielded eight modules; MEblack and MEturquoise had the highest correlation with NOA. Six genes in MEturquoise and RNF141 in MEblack were identified as key genes. qRT-PCR experiments validated the differential expression of key genes between NOA and control. Furthermore, RNF141 was regulated by the largest number of miRNAs.

**Conclusion:**

Our findings suggest that the significant change expression of key genes may be potential markers and therapeutic targets of NOA and may have some impact on the development of NOA.

## 1. Introduction

Infertility refers to the inability to carry out a clinical pregnancy after 12 months or more of unprotected sexual intercourse. According to worldwide statistics, infertility affects 10-15% of couples, and almost half of cases are caused by men [1]. Azoospermia is the most serious type of male infertility, which is mainly divided into obstructive azoospermia (OA) and nonobstructive azoospermia (NOA) [2]. Among them, nonobstructive azoospermia (NOA) accounts for 20% of infertility [3]. NOA is usually caused by the failure of spermatogenesis of unknown etiology and is a difficult problem in the field of male infertility [4]. The pathogenesis of NOA is unclear. Therefore, it is necessary to study the molecular mechanism of NOA and seek more potential therapeutic methods.

Despite systematic treatment options, the chances of successful clinical or surgical treatment for nonobstructive azoospermia are small [5]. Intracytoplasmic sperm injection (ICSI) is an effective method to treat severe male infertility as an assisted reproductive technology, but the success rate of NOA patients after ICSI treatment is about 36% [6]. Studies have found that spermatogenesis defects are significantly associated with decreased androgen levels [7]. Known causes of NOA include endocrine and chronic diseases (such as hypogonadism or diabetes) affecting the hypothalamic-pituitary-gonadal axis [8]. However, in patients with NOA, obvious genetic abnormalities are one of the causes of spermatogenic failure [9].

Spermatogenesis is an extremely complex process of cell differentiation involving 2,300 genes that regulate germ cell development and maturation [10]. The success of spermatogenesis and mature sperm release is related to the expression of large numbers of genes in spermatogenic cells [11]. The testis is an important organ determining human fertility and its endocrine status [12]. Many differences in testicular gene expression can be used to evaluate the underlying mechanism of spermatogenesis failure in NOA patients [13]. In addition, the evaluation of these gene transcripts can reflect the status of spermatogenesis in the corresponding testis and may be a potential therapeutic target.

In addition, microRNAs (miRNAs) posttranscriptionally regulated gene expression. Studies have shown that miRNAs play a crucial role in spermatogenesis [14]. It is suggested that miRNAs may mediate the development of spermatogenic cells by targeting the expression of mRNAs and participate in spermatogenesis and male infertility [15].

Weighted gene coexpression network analysis (WGCNA) is a widely used method to build coexpression pairwise correlation matrices [16]. Protein-protein interaction (PPI) network has also been frequently used to identify candidate genes for diseases [17]. There have been studies utilizing WGCNA and PPI networks together to screen potential target signals [18]. In this study, the transcriptome data of NOA patients in public databases were used to explore the molecular dysregulation mechanism and potential target genes.

## 2. Materials and Methods

### 2.1. Data Collection

The gene expression profiles of NOA and normal controls were collected from the Gene Expression Omnibus (GEO) database [19]. GSE45885 included gene expression array data of testicular biopsy samples from 27 human testicular biopsies in men with various nonobstructive azoospermias and 4 with normal spermatogenesis based on GPL6244. GSE145467 included gene expression array data of 20 testis samples (10 showing obstructive azoospermia and 10 samples showing nonobstructive azoospermia) based on GPL4133. Raw data were normalized with robust multiarray average (RMA) method [20].

### 2.2. Difference Analysis

Differential expression analysis was performed using the limma R package [21, 22] to identify differentially expressed genes (DEGs) between NOA and normal spermatogenesis. Genes with ∣log_2_(fold change) | >1 and *P* value < 0.05 in GSE45885 and ∣log_2_(fold change) | >2 and *P* value < 0.05 in GSE145467 were assigned as significantly different.

### 2.3. Biological Function

The Gene Ontology (GO) and Kyoto Encyclopedia of Genes and Genomes (KEGG) enrichment [23] was analyzed by using clusterProfiler R package [24–26] for DEGs. Screening threshold *P* value < 0.05 was considered significantly enriched.

### 2.4. Weighted Gene Coexpression Network Analysis (WGCNA)

The gene coexpression networks based on topological overlap were identified by WGCNA analytical method. The coexpression modules for DEGs of GSE45885 were constructed through WGCNA R package [16]. Eigengene expression patterns within each module are condensed into a “Module eigengene (ME).” The correlation between modules and clinical traits was calculated using Pearson correlation based on clinical information.

### 2.5. Protein-Protein Interaction (PPI) Network Analysis

The selected genes were performed through PPI network using the Search Tool for the Retrieval of Interacting Genes (STRING) database. The combined score > 0.6 was considered significant. The PPI network was visualized by Gephi software. The key genes were chosen based on their degree of connectivity with other genes. Visualization of the network of key genes is through Cytoscape software [27, 28].

### 2.6. Sample Collection

Peripheral blood samples from 5 NOA patients and 5 normal controls were collected from our hospital. All subjects read and signed the informed consent form. The study was approved by the ethics committee of our hospital.

### 2.7. Quantitative Real-Time Polymerase Chain Reaction (qRT-PCR)

The total RNA was isolated using TRIzol (Invitrogen, California, USA) from blood samples. Reverse transcription expression was performed using PrimeScript™ RT Master Mix (TaKaRa, Dalian, China). The qRT-PCR was carried out using the SYBR Green Master Mix (Invitrogen, California, USA) according to the manufacturer. The primer sequences of genes are shown in Table 1. Relative expression of genes was calculated using the 2^−ΔΔCT^ method [29]. Genes were normalized to GAPDH [30].

### 2.8. Statistical Analysis

Data analysis used SPSS 20.0 software. Data were presented as the mean ± standard deviations (SD) [31, 32]. Student's *t*-test was used to compare the differences between two groups. *P* value < 0.05 was considered statistically significant. The test level is *α* = 0.05 (two-sided).

## 3. Results

### 3.1. Abnormal Gene Expression in NOA Patients

**Figure 1:**
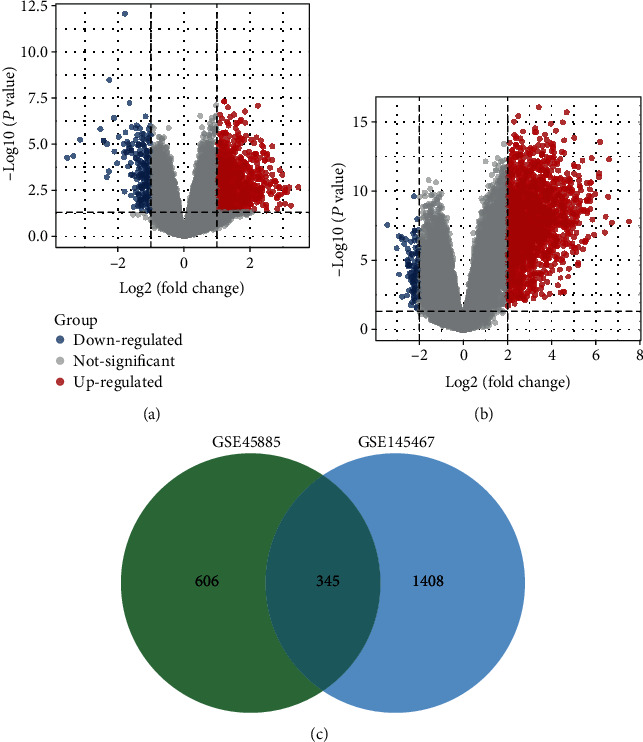
Differentially expressed genes of nonobstructive azoospermia. (a) The differentially expressed genes between NOA and control in GSE45885. The ∣log_2_(fold change) | >1 and *P* value < 0.05 were screening threshold. (b) The differentially expressed genes between NOA and control in GSE145467. The ∣log_2_(fold change) | >2 and *P* value < 0.05 were screening threshold. (c) The Venny map of two groups of differentially expressed genes. A total of 345 common genes were then identified.

To identify gene expression changes in NOA patients, we performed differential analysis of gene expression between NOA patients and normal spermatogenesis. A total of 951 significantly differentially expressed genes (DEGs) were found in GSE45885 (Figure 1(a), Table S1). Then, 1753 significantly differentially expressed genes were found in GSE145467 (Figure 1(b), Table S2). Of these, we found 345 common DEGs (Figure 1(c)). These genes may be significantly associated with NOA.

### 3.2. Biological Functions of NOA-Related Genes

**Figure 2:**
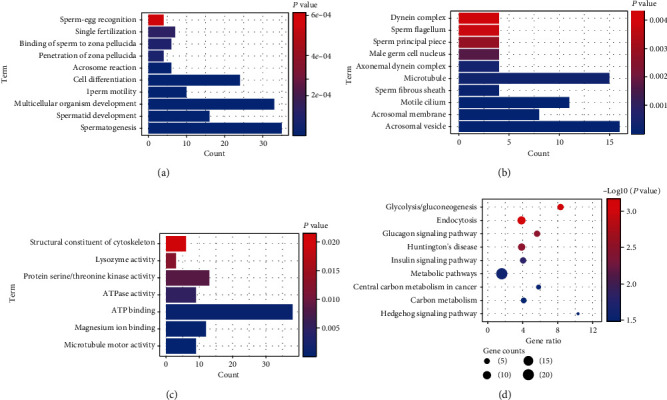
GO function and KEGG pathway of NOA-related genes. (a) The biological process of NOA-related gene enrichment. The longer the column, the greater the number of genes involved in this term. The redder the color, the higher the significance. (b) The cell composition of NOA gene enrichment. The longer the column, the greater the number of genes involved in this term. The redder the color, the higher the significance. (c) The molecular function of NOA-related gene enrichment. The longer the column, the greater the number of genes involved in this term. The redder the color, the higher the significance. (d) The KEGG pathway of NOA-related gene enrichment. The larger the circle, the greater counts of gene involved in this term. The redder the color, the higher the significance.

Through enrichment analysis of common DEGs, we found a lot of important terms in GO results. Biological processes (BP) mainly involved spermatogenesis, spermatid development, and sperm motility (Figure 2(a)). Cell composition (CC) mainly included acrosomal vesicle, sperm fibrous sheath, and sperm principal piece (Figure 2(b)). Molecular function (MF) mainly included microtubule motor activity, ATPase activity, and lysozyme activity (Figure 2(c)). In addition, we obtained 9 significantly enriched KEGG signaling pathways (Figure 2(d)), including the “insulin signaling pathway,” “metabolic pathways,” and “Hedgehog signaling pathway”.

### 3.3. Network Analysis of NOA-Related Genes

**Figure 3:**
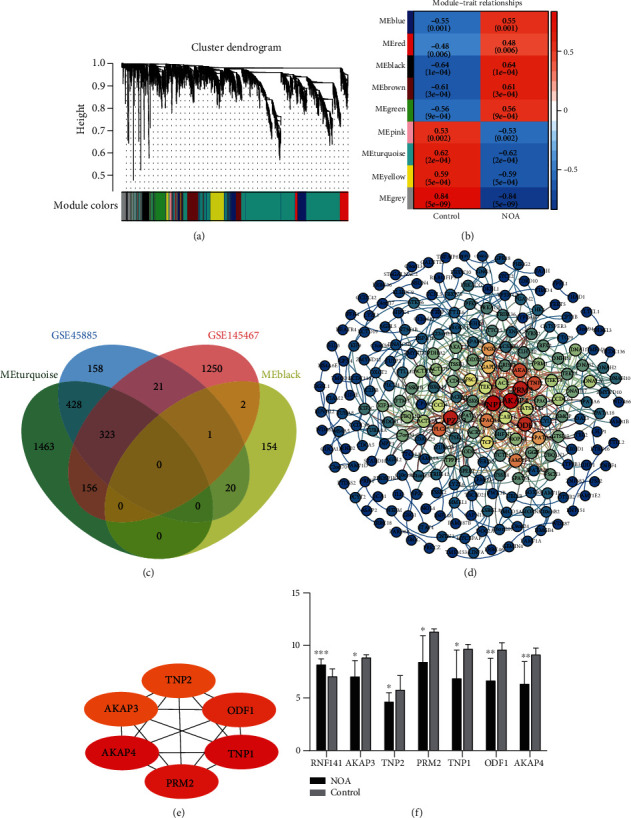
The WGCNA network of DEGs. (a) The DEGs of GSE45885 were clustered into eight coexpression modules. Different colors represent different modules. (b) Correlation between coexpression modules and clinical trait. Red is positive correlation and blue is negative correlation. (c) Intersection of important module genes with common genes. Different colors represent different groups. Intersection is then the intersection genes among groups. (d) PPI network of intersection genes between MEturquoise and common genes. The redder the color, the more connectivity of gene in the network. (e) The top 6 genes with the greatest connectivity in the PPI network. (f) The expression of key genes in NOA and controls of GSE45885. ^∗^*P* < 0.05, ^∗∗^*P* < 0.01, and ^∗∗∗^*P* < 0.001.

We performed WGCNA network analysis on DEGs of GSE45885. These genes were formed into eight coexpression modules by removing the grey module which without coexpression behavior (Figure 3(a)). Correlation analysis between module and trait showed that MEblack had the highest positive correlation with NOA and MEturquoise had the highest negative correlation with NOA (Figure 3(b)). To identify key genes that had important effects on NOA, we performed an intersection analysis between MEblack, MEturquoise, and common DEGs (Figure 3(c)). RNF141 was found in MEblack, and 323 intersecting genes were found in MEturquoise. Further, we constructed a PPI network for 323 genes (Figure 3(d)). The top six genes with the greatest connectivity were identified as key genes (AKAP3, AKAP4, TNP1, TNP2, ODF1, and PRM2) (Figure 3(e)), as well as RNF141. In GSE45885, RNF141 was highly expressed in NOA, while AKAP3, AKAP4, TNP1, TNP2, ODF1, and PRM2 were low expressed (Figure 3(f)).

### 3.4. miRNA Regulatory Network for Key Genes

**Figure 4:**
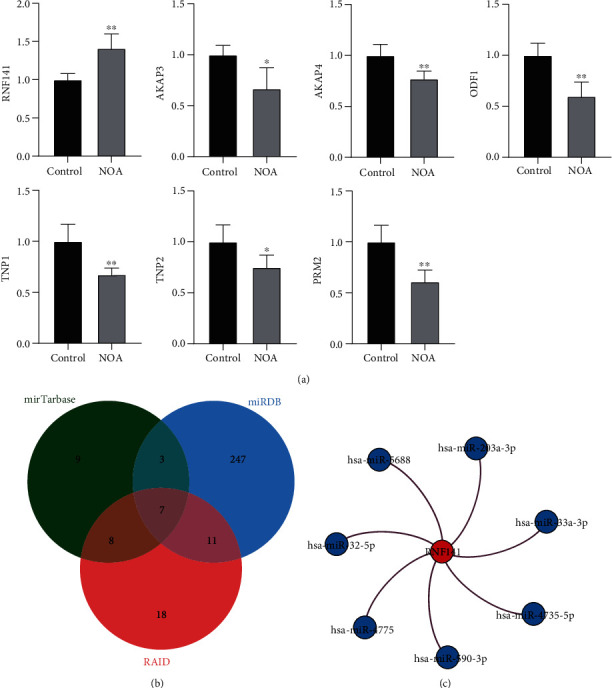
Validation experiment and regulatory network key genes. (a) The qRT-PCR experiments validated the differential expression of key genes between NOA and control for blood samples. ^∗^*P* < 0.05 and ^∗∗^*P* < 0.01. (b) Online prediction of miRNA regulators for key genes. Seven common miRNAs were found in the three prediction sites. (c) The regulatory network of seven common miRNAs to RNF141.

Through qRT-PCR experiments, we verified the differential expression of key genes between NOA patients and normal controls (Figure 4(a)). miRTarBase, miRDB, and RAID were used to predict the miRNA regulators of key genes, respectively. We obtained seven common miRNAs (hsa-miR-32-5p, hsa-miR-590-3p, hsa-miR-203a-3p, hsa-miR-4775, hsa-miR-4735-5p, hsa-miR-33a-3p, and hsa-miR-5688) (Figure 4(b)). Interestingly, these miRNAs all target RNF141 (Figure 4(c)).

## 4. Discussion

Thousands of genes have been involved in the establishment of male fertility potential, and many others have not yet been revealed [33]. Abnormal gene expression is important for understanding the etiology of male infertility. In this study, we identified gene expression changes in NOA patients and screened potential markers and molecular targets of NOA based on NOA-related gene expression profiles in the GEO database. To our knowledge, this is the first time that WGCNA and PPI network were together used to jointly screen the potential genes of NOA, and then, miRNA regulations for the key genes were further screened. Compared with other articles using these data [34, 35], we used multiple means of bioinformatics analysis to obtain more accurate potential target genes of NOA.

Severe impairment or absence of spermatogenesis is a characteristic of NOA in the biological functions involved by NOA-related DEGs [36]. Spermatogenesis is a complex process involving many transcription factors specific to cell type [37]. Normal spermatogenesis is dependent on Sertoli cells [38]. Genes related to the differentiation function of Sertoli cells are involved in glucose metabolism and insulin signaling [39]. One potential way to improve spermatogenesis is to optimize intratesticular testosterone (ITT) levels, and insulin-like factor 3 is a serum marker that may predict ITT [40]. Seminal plasma is a noncellular liquid component of semen, including substances related to metabolism [41]. Proteomic changes in seminal plasma of asthenozoospermia patients are mainly related to metabolism and energy production [42]. Arachidonic acid metabolism and inhibition of phospholipase may be associated with premature acrosome reaction [43]. The Hedgehog signaling pathway influences cell development, tissue homeostasis, cell proliferation and differentiation, and cell fate [44]. An increasing number of studies have shown that the Hedgehog signaling pathway in Sertoli cells is associated with spermatogenesis [45].

The positive correlation between MEblack and NOA was the highest, and the key gene RNF141 was expressed higher in NOA patients than in controls. Studies have shown that RNF141 is restrictively expressed in the testicular tissue of fertile men and involved in maintaining normal male fertility [46]. RNF141 is an E3 ubiquitin ligase that is recruited during oocyte maturation [47]. RNF141 is reported to be specifically present in acrosome and tail of mouse sperm acrosome [48]. In addition, RNF141 is regulated by multiple miRNAs and may play an important role in network regulation in NOA. miRNAs participate in the regulation of NOA through spermatogenesis and cell cycle [49]. Tyrosine phosphorylation of A-kinase-associated protein 3 (AKAP3) increases sperm binding to the zona pellucida [50]. AKAP3 and AKAP4 are the main components of fibrous sheath (FS) in the sperm tail [51, 52]. AKAP3 and AKAP4 play important benefit roles in sperm function, including regulation of sperm motility, sperm capacitation, and acrosome response [53]. AKAP4 is an ERK 1/2 substrate and a regulator of human sperm cAMP/PKA and PKC/ERK 1/2 pathways, which are associated with capacitation and acrosome reaction [54]. Transition proteins (TNP) are small proteins, the levels of TNP1 and TNP2 were significantly decreased in the testis and epididymis, and mild germ cell apoptosis was happened in both the testes and epididymis [55]. The reproductive potential of the TNP1- and TNP2-null mutant sperm in mice was reduced. [56]. Abnormal expression of TNP and PRM genes will lead to abnormal nuclear condensation, leading to male sterility [57]. Lack of protamine-2 (PRM2) can lead to severe membrane defects in spermatozoa, resulting in loss of motility and abnormal sperm head morphology and infertility [58]. PRM2 deficiency can trigger oxidative stress, leading to DNA damage, which leads to infertility [59]. It has been confirmed that the lack of outer dense fiber protein 1 (ODF1) is a marker and potential driver of idiopathic male infertility [60, 61]. Western blot showed that the expression level of ODF 1 in asthenozoospermia was indeed downregulated [62].

This study also has some limitations. First, our analytical data were derived from the GEO database, which lacked knowledge of the clinical data of the samples. Second, although we have validated key genes in clinical samples, more experimental data and clinical validation are needed for these genes to be applied. Another issue to consider is that spermatogenesis is a dynamic process, and this analysis provides information on gene expression and spermatogenesis status only at one point in time.

## 5. Conclusion

In conclusion, our preliminary findings suggested that RNF141, AKAP3, AKAP4, TNP1, TNP2, ODF1, and PRM2 were potential markers and therapeutic targets for NOA. They were associated with spermatogenesis and metabolic reactions. Given the potential impact of key genes on NOA, our findings suggest that further studies are necessary to confirm their role in NOA.

## Figures and Tables

**Table 1:** The primers of key genes for qRT-PCR.

Genes	Primers
GAPDH	F: 5′-CATGTTCGTCATGGGTGTGAA-3′
	R: 5′-GGCATGGACTGTGGTCATGAG-3′
RNF141	F: 5′-CCCATCCTCGGTCACATCTT-3′
	R: 5′-CCCCCTTCTCCTCTACGACAAC-3′
AKAP3	F: 5′-CAGGACTGGAAAATGGACACCT-3′
	R: 5′-TTTGTGTGGGTCTCCTGAGTTG-3′
AKAP4	F: 5′-TGATACTACAATGATGTCTGATGAT-3′
	R: 5′-GGAACTAGCAGCATCCTTGTAATCTTTATC-3′
TNP1	F: 5′-GCTGGATGCCAATCGC-3′
	R: 5′-GTCCCTTCTGTTCGGTTG-3′
TNP2	F: 5′-GTCCCTTCTTTCGGGTTG-3′
	R: 5′-TCAGTTGTACTCCGTCGTCGGGGAG-3′
ODF1	F: 5′-CCGCACTGAGTTGTCTTTTGG-3′
	R: 5′-GGGTGCATGTATAAGTCACACA-3′
PRM2	F: 5′-ATGGTTCGCTACCGAATGAGG-3′
	R: 5′-CTCCGCCTTCTGCATGACC-3′

## Data Availability

The corresponding data can be found in GSE45885 and GSE145467.

## Supplementary Materials

Supplementary Materials Table S1: the differentially expressed genes between NOA and normal spermatogenesis in GSE45885. Table S2: the differentially expressed genes between NOA and normal spermatogenesis in GSE145467.
